# Invadopodia, a Kingdom of Non-Receptor Tyrosine Kinases

**DOI:** 10.3390/cells10082037

**Published:** 2021-08-09

**Authors:** Trishna Saha, Hava Gil-Henn

**Affiliations:** Cell Migration and Invasion Laboratory, The Azrieli Faculty of Medicine, Bar-Ilan University, Safed 1311502, Israel; trishna.saha27@gmail.com

**Keywords:** invadopodia, non-receptor tyrosine kinases, cancer metastasis

## Abstract

Non-receptor tyrosine kinases (NRTKs) are crucial mediators of intracellular signaling and control a wide variety of processes such as cell division, morphogenesis, and motility. Aberrant NRTK-mediated tyrosine phosphorylation has been linked to various human disorders and diseases, among them cancer metastasis, to which no treatment presently exists. Invasive cancer cells leaving the primary tumor use invadopodia, feet-like structures which facilitate extracellular matrix (ECM) degradation and intravasation, to escape the primary tumor and disseminate into distant tissues and organs during metastasis. A major challenge in metastasis research is to elucidate the molecular mechanisms and signaling pathways underlying invadopodia regulation, as the general belief is that targeting these structures can potentially lead to the eradication of cancer metastasis. Non-receptor tyrosine kinases (NRTKs) play a central role in regulating invadopodia formation and function, but how they coordinate the signaling leading to these processes was not clear until recently. Here, we describe the major NRTKs that rule invadopodia and how they work in concert while keeping an accurate hierarchy to control tumor cell invasiveness and dissemination.

## 1. Introduction

Although primary tumors can be surgically removed and are usually responsible for only a small fraction of cancer-associated deaths, local invasion and/or metastatic dissemination of cancer cells to other tissues and organs are the primary causes of cancer mortality. Cancer-cell metastasis should therefore serve as a major focus in cancer research and a primary target for therapeutic intervention. To penetrate the basement membrane of the tumor epithelium and to intravasate through the vascular endothelium membrane into blood vessels, invasive cancer cells use invadopodia, actin-rich invasive foot-like processes with a matrix-degrading activity which converge signaling, cytoskeletal, adhesion, proteolysis, and membrane trafficking proteins and pathways [1,2]. Invadopodia-like structures were identified in several invasive cancer cell lines such as breast, head and neck, prostate, fibrosarcoma, and melanoma [3], and primary tumor cells obtained from patients with several different cancers exhibit the formation of invadopodia structures containing classical invadopodia markers that are associated with extracellular matrix (ECM) degradation activity [4,5,6]. Previous evidence demonstrates direct molecular links between invadopodia assembly and metastasis in mice models [7,8] and human patients [9]. Moreover, the existence of matrix-degrading invadopodia-like protrusions that form in response to a combination of microenvironmental cues was previously demonstrated in invasive primary tumor cells by live multi-photon microscopy in mice [10]. Invadopodia were first discovered and defined as surface protrusions with proteolytic activity 3 decades ago [11]. Since then, significant efforts have been allocated to elucidate the molecular mechanisms and signaling pathways that regulate their formation and function, as the general belief is that strategies aimed at disrupting invadopodia could form the basis of novel anti-metastasis therapies. Here, we describe the non-receptor tyrosine kinases (NRTKs) Pyk2, Src, Arg, and FAK, their invadopodial substrates, and their interplay in coordinating invadopodia formation and function and consequent cancer cell invasiveness.

## 2. The Secret Life of Invadopodia

The first stage in the invadopodium life cycle involves the assembly of the invadopod precursor structure and its stabilization. Invadopodium precursors are initiated by a combination of driver mutations within the tumor cells which drive tumor initiation and progression and stimuli such as growth factors, hypoxia, degraded ECM products, exosomes, and tumor cell–tumor-associated macrophage contact [12]. Following stimulation, the invadopodia proteins cortactin, cofilin, N-WASp, and the Arp2/3 complex form an initial core and associate with an actin filament. Tks5 arrives approximately 20 seconds later and anchors the precursor to the plasma membrane by binding to basal PI(3,4)P2 via its PX domain [13]. This is followed by the recruitment of SHIP2 to the precursor by lamellipodin [14], which de-phosphorylates PI(3,4,5)P3 into PI(3,4)P2. This de-phosphorylation event leads to a local increase in the levels of PI(3,4)P2, which enhances invadopodium precursor maturation and activation.

 The second stage of the invadopodium life cycle includes its maturation. During this stage, β1 integrin is recruited to the invadopodium precursors and is activated following ECM binding. While integrins are not involved in initiating the invadopodium precursors, they are essential for their stabilization and for triggering their maturation by co-activating non-receptor tyrosine kinases (NRTKs) [15]. NRTKs, which are co-activated by β1 integrin and epidermal growth factor receptor (EGFR) at invadopodium precursors, phosphorylate cortactin on two major tyrosines, Y421 and Y466. These phosphorylation events lead to actin polymerization in invadopodia by two synergistic pathways. First, phosphorylated cortactin recruits the adaptor protein Nck1, which binds to cortactin via its SH2 domain. Nck1 then binds and activates N-WASp, which recruits the Arp2/3 complex, and leads to actin polymerization in invadopodia [16]. Second, the focal adhesion protein talin is recruited to invadopodia in an integrin- and moesin-independent manner by binding to actin via its C-terminus I/LWEG motif. Following its arrival, talin binds to b1 integrin, enhancing the interaction between talin and moesin to recruit a moesin-NHE1 complex to invadopodia [17]. NHE1 increases the local intracellular pH at invadopodia and disrupts the inhibitory pH-dependent interaction between cortactin and cofilin, allowing cofilin to be released and activated [18]. Cofilin then severs F-actin to generate free actin barbed ends, which support dendritic nucleation by the cortactin-phosphorylation-mediated Nck1-N-WASp-Arp2/3 pathway. This actin polymerization event induces the invadopodium membrane protrusion. Phosphorylated cortactin also recruits the guanine-nucleotide exchange factor (GEF) Vav2, which promotes Rac3 activation and consequent actin polymerization in invadopodia, leading to invadopodia maturation [19]. Besides its role in enhancing actin polymerization in invadopodia, Rac3 was recently shown to integrate the adhesion process to ECM with its ability to degrade it via controlling Arf6-mediated vesicular trafficking [20].

In the last stage of the invadopodium life cycle, microtubules and intermediate filaments are recruited to invadopodia and cooperate with actin filaments to facilitate elongation of the invadopodium protrusion [21]. Shortly after, RhoA and CDC42 stimulate the association of IQGAP with the exocyst complex, which cooperates with endosomal WASH to promote MT1-MMP delivery to invadopodia [22]. Cortactin has an essential role in the formation of invadopodium precursors and their maturation and their activation. During the final stage of the invadopod life cycle, cortactin cooperates with Rab27a and coronin 1b to control the stability of cortical actin multivesicular late endosomes (MVEs) docking sites and exosome secretion. These exosomes are secreted into the extracellular environment and contain matrix metalloproteases (MMPs), enabling the tumor cell to degrade its surrounding ECM [23].

## 3. The Dual Face of Src Kinase

Viral Src (v-Src) was the first oncogene to be discovered, and its cellular counterpart (c-Src) is among the best-studied to date. Src is overexpressed and activated in various human tumors and has been linked to both cancer development and its progression to distant metastasis. In addition to increasing tumor proliferation, Src plays a crucial role in promoting motility and invasion, functions that contribute to tumor progression and metastasis [24].

Overexpression of wild-type or activated mutant Src in breast cancer cells or head-and-neck squamous cell carcinoma cells increases the number of invadopodium precursors, albeit having an overall shorter lifetime [25,26,27]. Inhibition of the Src family members by the kinase inhibitor PP2 reduces the number of invadopodium precursors and consequent ECM degradation [26]. However, specific knockdowns of Src do not change the number of precursors, but they significantly reduce matrix degradation [28]. Src is involved in invadopodium precursor formation and stabilization by regulating tyrosine kinase substrate 5 (Tks5) and activating the Abl-related gene (Arg) during invadopodium maturation and activation.

Tks adaptor proteins are essential regulators of various physiological and pathological processes such as cell migration and invasion, and cancer progression, by acting as scaffolds that bring membrane and cellular components in close proximity to structures such as podosomes and invadopodia. As their name implies, Tks proteins are substrates of tyrosine kinases and, in particular, Src kinase. Importantly, tyrosine phosphorylation of Tks5 by Src is essential for Tks5-mediated invadopodium precursor formation and consequent cancer cell invasion [29]. Tks5 has four main functions in invadopodia. First, as a scaffold protein, Tks5 anchors the invadopodium precursor to the invadopod plasma membrane, an event that stabilizes the precursor core complex and enables the following stages, which lead to its maturation [13]. Second, Tks5 also acts as a scaffold protein for the actin polymerization machinery by recruiting Nck1 and Nck2 and mediating downstream activation of N-WASp and Arp2/3 [30]. The third function of Tks5 in invadopodia is the facilitation of localized generation of reactive oxygen species (ROS). It was previously demonstrated that Tks5 adaptor proteins have structural similarities to the NADPH oxidase complex (NOX), which produces ROS, suggesting that one of their functional roles is to produce ROS in invadopodia. NOX-produced ROS leads to the inhibition of the active site of protein phosphatases, thereby enhancing localized tyrosine kinase signaling in invadopodia, which is essential for invadopodium precursor maturation and activation [29,31]. The fourth role of Tks5 in invadopodia is to promote the localization and activation of proteases in invadopodia. More specifically, Tks5 interacts with members of the ADAM family of proteases and can directly integrate proteases with the actin assembly machinery [32]. Tks5 also interacts with Rab40b to tether MMP-containing vesicles to invadopodia [33].

The nucleation-promoting factor (NPF) and actin-associated protein cortactin localize to invadopodia in invasive cancer cells, where cortactin regulates their formation and function. Significantly, tyrosine phosphorylation of cortactin is a crucial event that leads to the generation of free actin barbed ends and actin polymerization for efficient ECM degradation and in vivo metastatic dissemination [16]. Because cortactin was initially identified as an Src substrate, and Src promotes cortactin phosphorylation when overexpressed in cells and can promote actin polymerization in vitro via interaction with Nck1 and N-WASp, it has been inferred for many years that Src exclusively mediates cortactin tyrosine phosphorylation in invadopodia. However, it was previously demonstrated that although Src is involved in tyrosine phosphorylation of cortactin in invadopodium precursors and their consequent maturation and activation, this is completed indirectly by phosphorylating and activating Arg kinase, which directly binds and phosphorylates cortactin on two major tyrosines [28].

## 4. Grow or Go

Abl and its homolog Arg define a distinct family of NRTKs, which link diverse stimuli from cell-surface growth factor receptors and adhesion receptors to signaling pathways controlling various processes such as cell proliferation, cell survival, cell adhesion, migration, and invasion [34]. It was previously shown that Arg mediates EGF-induced cortactin tyrosine phosphorylation in invadopodia, leading to barbed end generation and consequent actin polymerization, ECM degradation, and matrix-proteolysis dependent breast tumor cell invasion. In addition, epistasis experiments demonstrated that both Arg and Src are required for downstream cortactin phosphorylation in invadopodia. However, since Arg kinase activity is critical for free actin barbed end generation and actin polymerization and Src cannot compensate for Arg knockdowns, it was suggested that Src acts upstream of Arg within the same signaling pathway as phosphorylate cortactin in invadopodia. Hence, Arg is a central kinase that phosphorylates cortactin in invadopodia, while Src regulates invadopodia by promoting Arg activation and additional independent pathways [28].

A mouse xenograft model containing Arg knockdown mammary tumors was used to examine whether Arg controls invadopodia-mediated breast cancer metastasis in vivo. This model has demonstrated that Arg knockdown in breast tumor cells significantly reduces tumor cell invasion, intravasation, and spontaneous metastasis to the lungs. This supports the model of an EGFR-Src-Arg-cortactin pathway for invadopodium maturation and activation and consequent tumor cell invasiveness. Surprisingly and unexpectedly, and despite the lower invasion from these tumors, primary tumors that developed in Arg knockdown mice were significantly larger than their control counterparts. Furthermore, examining gene expression profiles in Arg knockdown cells demonstrated the up-regulation of proliferation genes from the Ras-MAPK pathway together with down-regulation of invasion-associated genes. Arg, therefore, promotes invadopodia-mediated tumor cell invasiveness and dissemination while simultaneously inhibiting tumor growth and acts as a switch in metastatic cells that governs the decision to divide or invade, a model which was named “grow or go” [7,35]. According to this model, the proliferation and invasion states in a primary tumor cell are functionally coupled in an inverse relationship, in which invasive cancer cells shut their cell cycle while moving due to not being able to use their cytoskeleton in motility and cell division simultaneously. Because they do not maintain cell division and proliferation while moving and invading, these cells need to preserve their existing DNA during the metastatic process, which they accomplish by up-regulating DNA repair genes, which may also be related to the increased resistance of invasive cancer cells to cytotoxic therapy [36].

The involvement of Arg in in vivo breast cancer invasiveness and metastatic dissemination suggests that Arg inhibition may block invadopodia-mediated breast cancer metastasis. Indeed, the ABL kinase inhibitors imatinib, nilotinib, and GNF5, mainly used to treat leukemias, disrupt invadopodium precursor formation and cortactin phosphorylation-mediated invadopodium maturation, which leads to decreased actin polymerization, reduced ECM degradation, and impaired matrix-proteolysis dependent invasion of breast cancer cells. Accordingly, in vivo MMP activation, tumor cell invasion, and spontaneous lung metastasis are significantly impaired in tumor-bearing xenograft mice treated with either inhibitor. These data suggest that ABL kinase inhibitors may potentially be repurposed and used in combination with standard cytotoxic therapies to block breast cancer metastasis [37].

## 5. The Emperor of All Kinases

Up-regulation of proline-rich tyrosine kinase (Pyk2) has been observed in several invasive human tumors, including glioma, hepatocellular carcinoma, non-small-cell lung carcinoma, prostate cancer, and early and advanced breast cancer [38,39]. However, the mechanism by which it regulates tumor cell invasiveness was unclear until recently.

**Figure 1:**
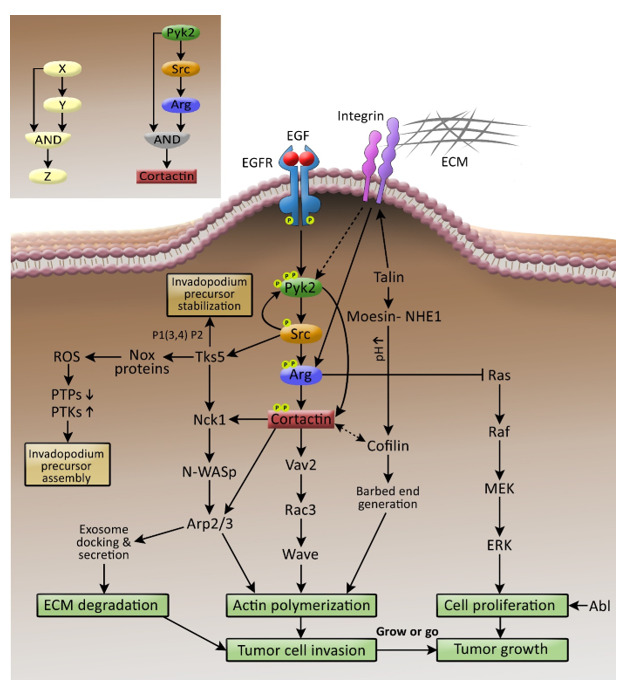
Pyk2-mediated signaling in invadopodia. Extracellular stimulation by EGFR and integrins leads to activation of Pyk2, which tyrosine phosphorylates cortactin both directly and indirectly by activating Src and Arg kinases. Activation of Src leads to invadopodium precursor assembly and stabilization by recruiting and phosphorylating Tks5. Complete tyrosine phosphorylation of cortactin leads to activation of several signaling pathways, which induce actin polymerization and ECM degradation and consequent invadopodia-mediated tumor cell invasion, intravasation, and metastatic dissemination. While promoting cell invasion and dissemination, Arg simultaneously inhibits the Ras-MAPK pathway, which induces cell proliferation and consequent tumor growth, acting as a switch in metastatic cancer cells that govern the decision to “grow or go” (divide or invade). Inset: the coherent type I feedforward loop with AND gating (left) parallels the Pyk2-Src-Arg-cortactin pathway (right). The different NRTKs, their substrates, and invadopodial roles are described in Table 1.

Using high-throughput protein array screening combined with bioinformatics and followed by biochemical and cellular validations, we identified cortactin as a novel substrate and interactor of Pyk2 in invadopodia. Significantly, Pyk2-mediated cortactin phosphorylation in invadopodia depends on both its kinase activity and the recruitment and activation of Src kinase. Moreover, epistasis experiments between Pyk2 and Arg demonstrated that both kinases are in the same signaling pathway, and they phosphorylate cortactin tyrosines in invadopodia. Thus, while Arg directly binds and phosphorylates cortactin, Pyk2 controls cortactin tyrosine phosphorylation directly by binding and phosphorylating cortactin and indirectly by recruiting and activating Src kinase, which activates Arg to phosphorylate cortactin in invadopodia [40] (Figure 1).

In systems biology terms, the invadopodial Pyk2-mediated signaling pathway is considered as a coherent type I feedforward loop with AND gating [41], where protein X (Pyk2) activates protein Z (cortactin) both directly and indirectly via protein Y (Arg) (Figure 1, inset). This type of network motif is characterized by both persistence, which enables prolonged signal duration, and redundancy, which allows signaling networks to withstand perturbations that can alter input–output relationships. This type of motif also provides a safety net for activation of critical processes essential for cell survival and homeostasis and ensures that cells respond only to stimuli of appropriate duration and magnitude. For example, invasive cancer cells leaving the primary tumors prepare for a long and risky journey within the body, from which there is no way back. To survive this journey and reach new environments where they can establish a secondary colony, a prolonged and persistent signal that can withstand perturbations is necessary [42,43]. The coherent type I feedforward loop mediated by the Pyk2-Src-Arg axis in invadopodia enables both characteristics. Moreover, because two phosphorylation events of cortactin by both Arg and Pyk2 are necessary, the redundancy in this system prevents activation of cortactin if phosphorylated on one site only or by one kinase only. Whether Arg and Pyk2 each phosphorylate cortactin on one specific tyrosine or both on the same tyrosine residue is a subject for future investigation.

Notably, high co-expression of Pyk2, Arg, and cortactin is correlated with a significantly higher metastatic potential of human breast tumors, suggesting that this pathway is essential for breast cancer invasiveness and dissemination and that a gene signature of this pathway could potentially be used as a strategy for predicting the metastatic potential of breast tumors. Moreover, a network interaction map of invadopodia-associated proteins has demonstrated a central role for the EGFR-Pyk-Src-Arg-cortactin invadopodial pathway, in which repurposing of existent inhibitors could be applied for blocking breast cancer metastasis [44].

A novel mechanism for activation of Pyk2 in invadopodia by calcium has recently been described. Pyk2 is a Ca2+/calmodulin-dependent kinase [45]. The binding of Ca2+/calmodulin to the autoinhibitory FERM domain leads to dimerization of Pyk2, which promotes its autophosphorylation and activation [46]. Stromal interacting molecule 1 (STIM1) and Orai1-mediated store-operated Ca^2+^ entry (SOCE) constitute the major calcium influx in most electrically non-excitable cells. Using the Orai1 biosensor that reports sub-plasmalemmal Ca^2+^ signals, Lu et al. have demonstrated that calcium glows arising from STIM-Orai1 appear locally at invadopodia of invading melanoma cells. These calcium glows lead to Ca^2+^/calmodulin-mediated activation of Pyk2, which initiates the SOCE-Pyk2-Src signaling cascade required for invadopodium precursor formation and subsequent tumor cell invasion [47].

## 6. Migrate or Invade: A Game of Thrones

Pyk2 and its homologous focal adhesion kinase (FAK) define a distinct family of NRTKs with high sequence and structural similarity and a common activation mechanism. FAK plays a critical role in cell motility in various cell types, where it coordinates lamellipodial protrusions and the turnover of adhesion complexes. Increased expression of FAK has been detected in metastatic tumors of the breast, prostate, colon, and brain, and mammary epithelial-specific disruption of FAK blocks mammary carcinoma progression in a genetic mouse model [50,51]. Another publication reported that mammary-specific deletion of FAK mainly affects primary tumor development and reduces the number and size of lung metastases [52]. However, it is unclear whether the lower frequency of metastases in these mice results from fewer tumor cells that escape from the primary tumor or because of an inherent defect in their locomotion and invasion.

Along the same lines, a previous publication suggested that FAK indirectly regulates invadopodium precursor formation and activation by sequestering Src away from invadopodia and into focal adhesions. By doing so, FAK controls the Src-dependent tyrosine phosphorylation balance between invadopodia and focal adhesions in invasive breast cancer cells [26]. Knockdown of FAK in mammary tumor cells leads to increased formation of invadopodium precursors, which is mediated by Src localization to the invadopodia. However, the identity of the protein that localizes Src to invadopodium precursors was unknown until recently. Using fluorescently tagged Src and breast cancer cells knocked down for either Pyk2 or FAK, it was demonstrated that while FAK recruits Src to focal adhesions, as was previously documented, Pyk2 is the kinase that controls the recruitment and localization of Src to invadopodia [40]. These data imply that Pyk2 and FAK coordinate the spatial regulation of Src kinase and consequently control invadopodium assembly and maturation. While Pyk2 regulates invasiveness by controlling cortactin tyrosine phosphorylation-dependent invadopodia maturation and consequent actin polymerization and ECM degradation, FAK controls invasiveness of tumor cells by controlling Src kinase-dependent focal-adhesion mediated motility. Coordination of both Pyk2-mediated invasion and FAK-mediated migration is necessary for breast cancer cell invasiveness and consequent metastatic dissemination [53].

Several publications have demonstrated the molecular interactions of Pyk2 with Src in regulating tumor cell motility and invasion. Overexpression of Pyk2 facilitates hepatocellular carcinoma (HCC) cell invasiveness by up-regulating phosphorylation of Src, ERK1/2, and MEK1/2 [54], and both Pyk2 and FAK interact with Src and p130Cas to regulate breast cancer motility and invasion [55]. However, the molecular mechanism by which Pyk2 recruits Src into invadopodium precursors to regulate their formation and maturation was not reported before.

Using a combination of multi-photon microscopy of mammary tumors in live mice and support vector machine algorithms, it was demonstrated that single invasive mammary carcinoma cells could move within the primary tumor using either focal-adhesion mediated fast motility on ECM fibers or invadopodia- and matrix-proteolysis mediated slow locomotion. The type of motility that invasive breast tumor cells use is determined by a combination of local extracellular cues present in the tumor microenvironment, such as local collagen fiber density, tumor cell density, tumor-associated macrophage density, the number and size of blood vessels, and the relative distance of the invasive cell from a tumor-associated blood vessel [10]. We suggest that while the switch between the two motility types of the invasive cancer cell is triggered by extracellular microenvironmental cues, Pyk2 and FAK are responsible for the intracellular integration and processing of these signals. Thus, the type of motility that the invasive cancer cell will activate will be the sum of these local microenvironmental signals.

## 7. The Other Side of The Coin

Reversible phosphorylation of tyrosine residues in proteins plays a central role in the regulation of various cellular processes such as cell cycle and cell division, development and morphogenesis, and cell motility, and is mediated by the opposing actions of protein tyrosine kinases (PTKs) and protein tyrosine phosphatases (PTPs). Although PTKs and PTPs are structurally and functionally different, they jointly regulate the phosphorylation of their common substrates [56]. Aberrant tyrosine phosphorylation of proteins that results from imbalanced activities of PTKs and PTPs may lead to various human disorders and diseases, among them cancer [57].

Cytoplasmic PTP1B is essential for both invadopodium precursor formation and activation and invadopodia turnover, and regulates these processes through direct and indirect mechanisms. Specifically, the calcium-dependent protease calpain 2 is activated following EGFR and/or integrin activation and cleaves PTP1B at a region near the C-terminus, enhancing PTP1B phosphatase activity. Activated PTP1B removes the inhibitory phosphate from Y529 of the invadopodial Src, which leads to both invadopodium precursor assembly and maturation. Calpain 2 also functions downstream of Src kinase to regulate the disassembly of cortactin from mature invadopodia, and consequently, the disassembly of the invadopodium structure [58].

Another substrate of PTP1B in invadopodia is cortactin. More specifically, PTP1B counteracts the activity of NRTKs, which phosphorylate cortactin on tyrosine 421, an event that leads to invadopodia activation. The actin barbed end capping protein isoform Mena^INV^ is recruited to invadopodium precursors by lamellipodin just after its initial assembly and stabilization at the plasma membrane and maintains tyrosine phosphorylation of cortactin at Y421 by displacing PTP1B from the precursors, thereby preventing PTP1B from de-phosphorylating cortactin and enhancing precursor maturation and activation [59].

## 8. The Hitchhiker

Hepatitis C virus (HCV) is a significant risk factor for developing HCC and leads to an aggressive and invasive disease with very low patient survival rates. The poor prognosis rates in HCC are usually a result of intrahepatic metastasis caused by the invasion of cancer cells from the primary tumor into other liver parts or by their dissemination via the portal vein. Dissemination into regions outside of the liver may also occur in the early stages of HCC due to aggressive treatment. The most effective treatment for early localized tumors is surgery, while sorafenib, a multi-kinase inhibitor, extends overall survival and delays tumor progression by a few months only in patients with advanced HCC [60,61]

While a strong association between chronic infection with HCV and the development of metastatic HCC is well established, the mechanism by which HCV enhances invasiveness and metastasis of HCC was not known until recently. By integrating the transcriptome with a functional kinome screen, we have recently shown that HCV infection enhances invadopodium precursor formation and activation by combining enhanced cytoskeletal, invasion, and invadopodia gene expression with the stimulation of kinase signaling [62].

Importantly, HCV enters the liver host cell by using EGFR as a coreceptor. Once the virus has penetrated the host cell, it maintains constant activation of EGFR and enhances invadopodia activation by activating the viral NS3/4A protein, which inhibits TC-PTP, thereby preventing de-phosphorylation of the EGFR receptor. Virus-activated EGFR then transmits signals to NRTKs and other invadopodial proteins, which lead to the maturation and activation of invadopodia in the host cell. In addition, HCV also misregulates chromatin organization and consequently enhances the transcription of invadopodia-associated genes, such as cortactin, MT1-MMP and EGFR ligands, such as HB-EGF and BTC, which support sustained activation of the receptor in an autocrine loop. A combination of these HCV-induced mechanisms leads to invadopodia-mediated cancer cell invasiveness and consequently intra- and extra-hepatic dissemination. Interestingly, kinome and transcriptome analysis revealed that all components of the EGFR-Pyk2-Src-Arg-cortactin axis are activated following HCV infection, suggesting that this pathway may be a general mechanism for invadopodia-mediated invasiveness of cancer cells. Moreover, examination of the invadopodia-associated gene signature in HCV-mediated HCC tumors has revealed that the gene expression pattern significantly correlates with poor patient prognosis and survival, supporting the essential role of invadopodia in promoting HCV-mediated invasiveness of HCC tumor cells [62].

As part of its life cycle, which relies entirely on the host cell infrastructure, HCV modifies signaling pathways involved in proliferation, cell survival, and cytoskeleton-mediated vesicular transport. Invasiveness and metastasis are not essential for the HCV life cycle, and the reason for inducing these processes by the virus within the host tumor cell is unclear. We suggest that by enhancing invadopodia-mediated dissemination of HCC tumor cells to new regions away from the primary tumor, HCV gains a new environment where, unlike the crowded primary tumor environment, the host cell no longer has to compete with other tumor cells for nutrients and oxygen, and where the virus can infect new, healthy liver cells to enhance its replication and production.

## 9. Concluding Remarks and Future Directions

A significant correlation exists between invadopodia-associated genes and tumor cell dissemination [44,62], suggesting that invadopodia could be used as a prognostic marker for cancer metastasis. This may spare many patients at low risk for metastatic disease from undergoing unnecessary and potentially dangerous treatments while identifying other patients with lower-grade tumors that may be highly metastatic. Moreover, understanding the molecular mechanisms and signaling pathways that govern the formation and function of invadopodia could provide insights into the biology, regulation, and potential therapeutic approaches to cancer metastasis.

The importance of targeting invadopodia in anti-metastasis treatment is apparent considering that invasion and intravasation of tumor cells from the primary, secondary, and tertiary tumors are crucial steps in the systematic dissemination of cancer and that these processes continue even after resection of the primary tumor. Because tumor cell dissemination and tumor growth are not always linked [7,63,64], such inhibitors are not expected to affect primary tumor growth. Nevertheless, such inhibitors may effectively inhibit the metastatic spread of highly invasive tumors when used in combination with cytotoxic treatments such as chemotherapy and radiotherapy, which complement anti-metastasis treatment by inhibiting proliferation and survival of cells in the primary tumor and cells that may have succeeded in escaping the primary tumor and seeding metastases in distant sites. Moreover, because invadopodia are not essential for cell viability, anti-invadopodia-based therapies are not expected to affect crucial cellular processes and may have fewer side effects than conventional cancer therapies.

NRTKs in the Pyk2-Src-Arg-cortactin cascade present excellent targets for anti-invadopodia-mediated metastasis treatment. Indeed, while inhibitors of Arg have proved to be efficient in inhibiting invasiveness and metastasis of human breast tumor cells in immunodeficient mice [37], no specific inhibitor for Pyk2 that is potent in vivo currently exists. Moreover, the interplay between Pyk2 and FAK in controlling invadopodia formation and function may suggest that inhibiting both kinases may be a more efficient strategy for blocking cancer metastasis.

While significant knowledge on invadopodia formation, regulation, and signaling had been accumulated since their first discovery and definition approximately 3 decades ago, a major challenge of bench-to-bedside application of these findings still exists and should become the primary focus of cancer metastasis research within the coming years.

## Figures and Tables

**Table 1:** Major NRTKs and their targets in the Pyk2-Src-Arg-cortactin invadopodial pathway.

NRTK	Target	Role in Invadopodia	Reference
Pyk2	Src	Pyk2 recruits and activates Src in invadopodia.	[40]
	Cortactin	Pyk2 tyrosine phosphorylates cortactin both directly and indirectly by recruiting and activating Src, which activates Arg to phosphorylate cortactin.	[40]
FAK	Src	FAK indirectly regulates invadopodium precursor formation and activation by sequestering Src to focal adhesions and away from invadopodia.	[26]
Src	Tks5	Src tyrosine phosphorylates Tks5 to regulate invadopodium precursor formation and stabilization.	[30,48,49]
	Arg	Src phosphorylates and activates Arg, which phosphorylates cortactin during invadopodium maturation and activation	[28]
Arg	Cortactin	Arg tyrosine phosphorylates cortactin in invadopodia, leading to invadopodium maturation and ECM degradation.	[28]
	Ras	Arg inhibits the Ras-MAPK pathway to block tumor cell proliferation.	[7]

## Data Availability

Not applicable.
